# The Complex Interaction between P53 and miRNAs Joins New Awareness in Physiological Stress Responses

**DOI:** 10.3390/cells11101631

**Published:** 2022-05-13

**Authors:** Camilla Capaccia, Silvana Diverio, Danilo Zampini, Gabriella Guelfi

**Affiliations:** 1 Department of Veterinary Medicine, Università degli Studi di Perugia, Via San Costanzo 4, 0126 Perugia, Italy; camilla.capaccia@unipg.it (C.C.); danilo.zampini@unipg.it (D.Z.); 2 Laboratory of Ethology and Animal Welfare (LEBA), Department of Veterinary Medicine, Università degli Studi di Perugia, Via San Costanzo 4, 0126 Perugia, Italy; silvana.diverio@unipg.it

**Keywords:** P53, miRNA, physiological stress, stress outcomes, epigenetics, gene expression

## Abstract

This review emphasizes the important role of cross-talk between P53 and microRNAs in physiological stress signaling. P53 responds to stress in a variety of ways ranging from activating survival-promotion pathways to triggering programmed cell death to eliminate damaged cells. In physiological stress generated by any external or internal condition that challenges cell homeostasis, P53 exerts its function as a transcription factor for target genes or by regulating the expression and maturation of a class of small non-coding RNA molecules (miRNAs). The miRNAs control the level of P53 through direct control of P53 or through indirect control of P53 by targeting its regulators (such as MDMs). In turn, P53 controls the expression level of miRNAs targeted by P53 through the regulation of their transcription or biogenesis. This elaborate regulatory scheme emphasizes the relevance of miRNAs in the P53 network and vice versa.

## 1. P53 Is a Key Regulator of Cell Survival Pathways

Physiological cellular stress can be described as the outcome of the set of changes, related to the environment, that significantly disrupts cellular homeostasis by causing reversible damage to macromolecules such as proteins, DNA, RNA, and lipids. Cells in response to stress activate mechanisms such as growth arrest, repair or elimination of damaged macromolecules, changes in gene expression programs, not only to restore cellular homeostasis but also to enable adaptive homeostasis. Adaptive homeostasis enables biological systems to continually make short-term adjustments for optimal performance. If cellular damage is excessive, as caused by high doses of a stressor, programmed cell death (apoptosis) is triggered [1].

The tumor suppressor P53 protein, encoded by the *TP53* gene, and its signaling pathway composed of hundreds of genes play a central role in maintaining homeostatic functions and adaptation to environmental fluctuations. The *TP53* gene encodes a tumor-suppressive protein, activated by DNA damage, and also by other types of cellular stress. The functionally active P53 limits tumor development by limiting cell propagation into adverse conditions such as DNA damage, oncogene expression, and cellular stress. Even if growing normally, mice lacking P53 develop cancer before six months of age; however, there is growing evidence that P53 also contributes to pathologies other than cancer [2].

P53 is highly inducible by many stress signals, such as genotoxic stress, starvation, hypoxia, and oncogene activation. In non-stressed cells, the level of P53 is low, but during sustained stress signals, P53 drives irreversible programs of apoptosis or senescence to cull irreparably damaged or malignant cells for the ultimate benefit of the organism. In the event of low-level stress, P53 induces pro-survival homeostatic responses to protect genome integrity and to maintain viability in cells that suffer limited, reparable damage. The capability of P53 to coordinate multiple cell signaling pathways and determine cell fate has been the focus of numerous studies for many years [2].

**Figure 1:**
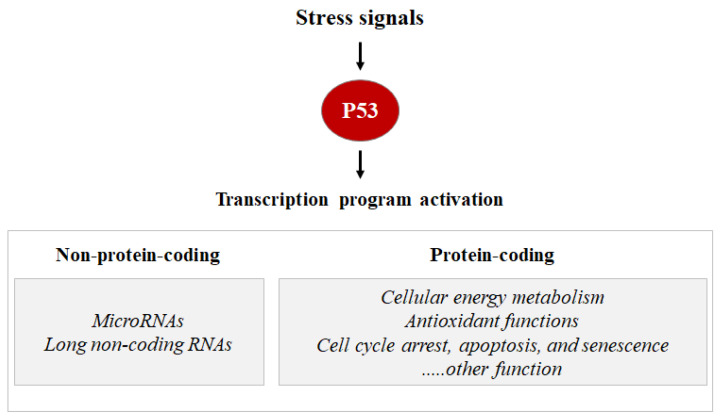
Stress signals can lead to P53 activation. Consequentially, the P53 can induce the transcription activation of coding proteins, ranging from the induction of cell cycle metabolism control, antioxidant functions apoptosis, senescence, cell cycle arrest, and DNA repair, or the P53 can induce the transcription activation of non-coding proteins such as microRNA and long non-coding RNAs.

As a transcription factor, the P53 protein mainly exerts its functions through transcriptional regulation by activating or repressing several functional clusters of genes. In response to a wide variety of intracellular and extracellular stress signals, P53 is mainly activated through post-translational modifications, leading to an increase in P53 half-life and its accumulation inside the cells. Thus, the transcription factor P53 binds to P53 response elements (P53RE) present in the early intron promoters of P53 target genes and induces the transcription of protein-coding or non-coding genes (microRNAs, etc.) involved in P53-mediated responses (Figure 1).

In addition to transcription activation, P53 can also repress gene expression. The P53-dependent repression can occur directly through the binding of P53 to specific P53RE, or by recruiting co-repressors such as SIN3A. The P53–SIN3A complex targets HDAC1 (Histone Deacetylase 1) to P53-repressor promoters to create a chromatin environment that is not conducive to transcription. In addition, P53 can lock the binding sites for transcriptional activators that promote gene expression or the repression can occur through P53-dependent activation of non-coding RNAs that downregulate gene expression [3].

**Figure 2:**
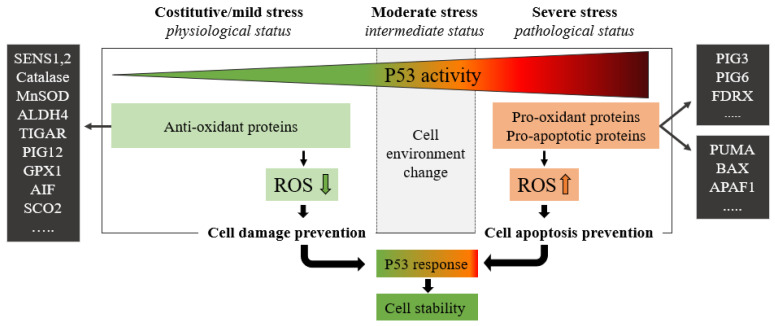
The green zone represents a low-risk state of constitutive/mild stress in which there is a homeostatic recovery. The dashed line represents the critical level of P53 activity; in this area, P53 attempts to ensure genome stability and cellular homeostasis by modulating P53-regulated genes. As the expression of P53 increases, the polychromatic triangle (**top**) changes from green to red, and cell damage risk increases due to the P53 expression level increment. During constitutive/mild stress, moderate stress, and severe stress, the cellular environment changes, and anti-oxidant or pro-oxidant proteins vary the ROS expressions with respect to the stress level. In mild stress, anti-oxidant proteins lower the level of ROS preventing cell damage. Conversely, in severe stress states, a high level of pro-oxidant and pro-apoptotic proteins increases ROS levels by inducing cell damage and apoptosis. ROS levels depend on P53 expression which in turn is stimulated by high ROS levels.

P53 protein plays a critical role in maintaining cellular energy metabolism by controlling oxidative phosphorylation and downregulation of glycolysis. Loss of P53 results in the deficiency of mitochondrial oxidative phosphorylation and enhanced glycolysis in both cultured cells and mouse models [4]. Another important function of P53 is to improve antioxidant defense by reducing ROS levels. To exert its antioxidant function, P53 induces a group of antioxidant genes, including a stress-inducible protein that regulates metabolism such as SESTRINs (SENS1, 2), TIGAR, GPX1, ALDH4, GLS2, and MnSOD, especially under conditions of non-stress or constitutive/mild stress, to lower ROS levels and prevent DNA damage [4]. Remarkably, in addition to antioxidant roles, P53 may exert a prooxidant effect via the upregulation of prooxidant genes, including *PIG3, PIG6, FDXR*, *BAX*, and *PUMA* [4]. Exposure of cells to high levels of ROS leads to oxidative stresses, which induce the P53 response (Figure 2). 

In response to prolonged or severe stress signals that induce irreparable damage, P53 drives irreversible apoptosis or senescence programs. P53 triggers apoptosis by the transcriptional induction of components of both the extrinsic and intrinsic death pathways, including BAX, FAS, NOXA, and PUMA [3]. In other cases, P53 responds to severe stress not with apoptosis but by inducing cellular senescence. Senescence is likely to result from the transcriptional activation of target genes such as *CDKN1A*, plasminogen activator inhibitor 1 (*PAI1*), and *PML* [3].

## 2. P53 Post-Transcriptional and Post-Translational Regulation

**Figure 3:**
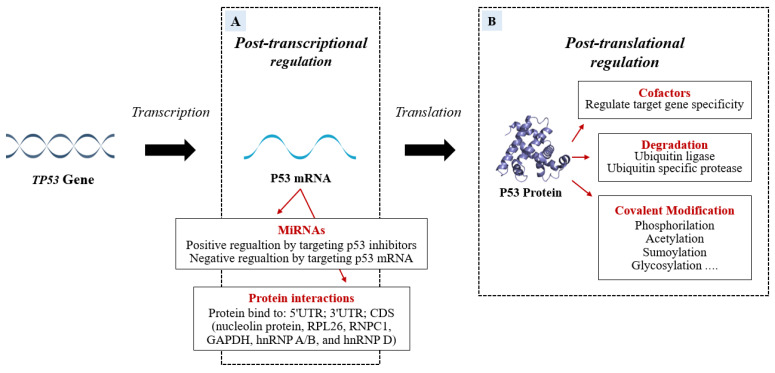
The P53 mRNA can be regulated post-transcriptionally (**A**) by miRNAs and RNA-binding proteins (RBPs) which modify the P53 transcriptional program in a stimulus-specific manner. The P53 miRNA regulation can be positive if miRNAs bind P53 inhibitors or can be negative if the miRNAs target is P53 mRNA. In addition to miRNA action, the P53 post-transcriptional program is also co-regulated, both negatively and positively, by RBPs that bind 5′UTR; 3′UTR; CDS of *TP53*. Some proteins modify P53 post-translationally. The figure (**B**) shows the P53-modifications of specific amino acid residues divided into Covalent Modification, Ubiquitination, and cofactors.

Several factors are able to specifically bind P53 mRNA or P53 protein to regulate P53 transcription or translation (Figure 3).

Important post-transcriptional regulators bind to P53 mRNA and regulate the protein levels to protect undamaged cells from the devastating consequences of abundant P53 and at the same time to ensure that damaged cells do not escape surveillance [5]. Recently, miRNAs have emerged as post-transcriptional regulators of P53. MiRNAs can be involved in physiological or pathological processes by regulating the abundance and/or activity of P53 (the role of miRNAs will be discussed in detail below). In response to cellular stresses, the interplay of P53 and post-translational modifications fine-tunes P53 activity to further prevent cellular harm. The post-translational level of the protein is primarily regulated by modifications such as ubiquitination, phosphorylation, acetylation, methylation, glycosylation, neddylation, and sumoylation.

The P53-ubiquitin ligase forms an autoregulatory loop resulting in lower P53 activity. Murine Double Minute 2 protein (MDM2), an E3 ubiquitin ligase, is the most important negative regulator of P53. Not only MDM2 but also other E3-ligases (such as COP1, PIRH2, and ARF-BP1) contribute to the control of P53 levels. MDM2 keeps at a low-level P53 in non-stress conditions through polyubiquitylation and proteasomal degradation. MDM2 binds to the transactivation domain of P53 and ubiquitylates its six C-terminal lysines, targeting it for proteasomal degradation. Together, P53 and MDM2 function in a negative feedback loop, P53, by binding to the *MDM2* promoter, modulates the expression of MDM2 which in turn promotes the degradation of P53 and quenches cellular P53 activity [6].

**Figure 4:**
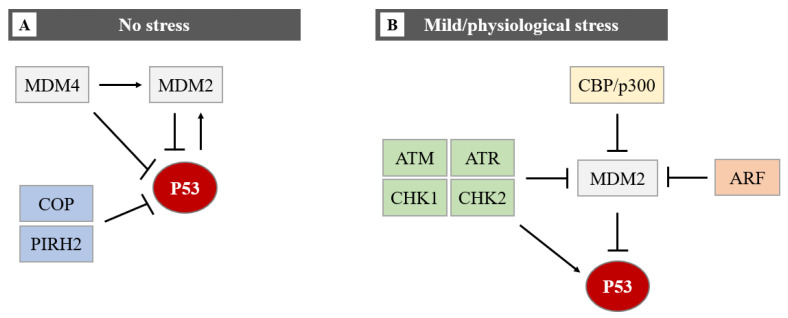
The regulation of the P53 protein level in cells in no stress and mild/physiological stress. During no stress (**A**), E3-ubiquitin ligases (MDM2, COP, and PIRH2) inhibit P53 activity through polyubiquitination and proteasome degradation. The function of P53 and MDM2 is regulated by a negative feedback loop. MDM4 can bind directly to P53 by repressing P53 transcriptional activity, or it can promote MDM2-mediated degradation of P53. The MDM2-P53 association promotes the degradation of P53 because physiological degradation of P53 is ubiquitin-dependent and MDM2 acts as a P53-specific E3 ubiquitin ligase. In conclusion, as MDM2 expression is positively regulated by P53, MDM2 drives P53 proteasomal degradation, it becomes apparent that MDM2 and P53 form an autoregulatory loop. In this loop, MDM2 restrains P53 levels under normal physiological conditions. Under mild/physiological stress states (**B**), MDM2 levels are kept low by ATM, ATR, CHK1 and CHK2, ARF, and CBP/p300. The low level of MDM2 induces the expression of P53. In the presence of mild/physiological stress, the level of these proteins increases.

In homeostatic conditions, P53 induces the transcription of the *MDM2* gene maintaining low levels of P53 protein. However, upon cellular stress events, P53 protein levels rise due to a disruption of P53-mediated MDM2 transcription [7,8]. This negative feedback loop helps maintain the low cellular level of P53 under normal conditions and limits the duration and potency of the P53 activation under stress conditions [9]. Induction of P53 involves uncoupling it from its negative regulators, principally MDM2 and the related protein MDM4 (also known as MDMX). MDM4 has been identified as a protein sharing structural homology with MDM2, especially in the N-terminal P53 binding domain and both proteins have a role in maintaining low levels of P53 in normal cells. MDM4 suppresses P53 transcriptional activity by binding to its transactivation domain [10]. In addition, MDM4 downregulates P53 by enhancing MDM2-mediated degradation (Figure 4).

The link between circular RNA (circRNAs) and P53 represents a further regulatory element of P53 whose implications may deserve great interest. CircRNAs are single-stranded non-coding RNAs, strongly involved in the regulation of P53 although there is information on their role in physiological stress. The circRNAs deriving from exons are generated from precursor mRNA mainly via a back-splicing process, in which the 3 ‘end of the exon ligates to the 5’ ends of its own forming a closed structure. The circRNAs can exert their function by binding and interacting with P53, to regulate the transcription process, acting with a mechanism similar to that of the ubiquitin ligase E3, MDM2, and COP1, resulting in the inhibition of P53 [11].

## 3. P53 Transcriptional Activation, and miRNA-mRNA Regulatory Network

**Figure 5:**
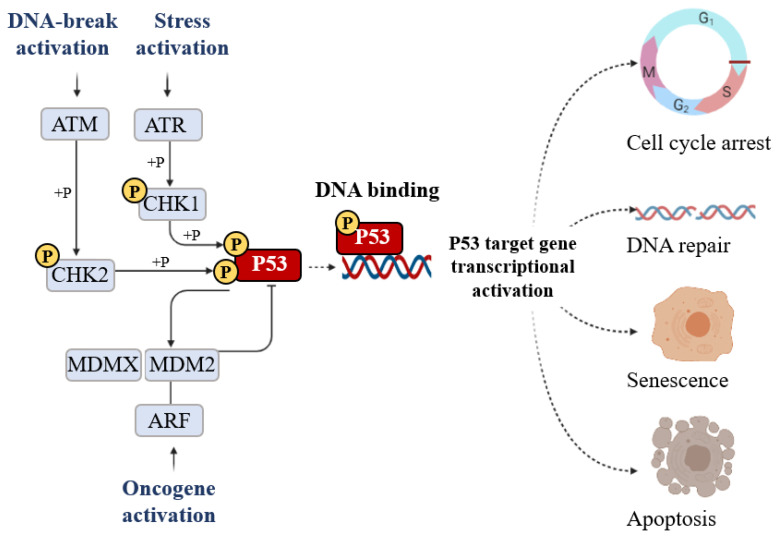
The signal that induces P53 activation is its phosphorylation (+P) which is triggered by DNA breakage activation (ATM), stress activation (ATR), and oncogene activation (ARF). Phosphorylation stabilizes P53 and promotes DNA binding. DNA-bound P53 then recruits the transcriptional machinery to activate the transcription of P53 target genes able of inducing cell cycle arrest, DNA repair, senescence, and apoptosis.

The classical model for P53 activation consists of three sequential activating steps: stress-induced activation mediated by phosphorylation sequence-specific DNA binding, and transcriptional activation of the P53 target gene (Figure 5).

Post-translational modification is the major mechanism of P53 regulation and phosphorylation events strongly affect the transactivation function of P53. Following stress, P53 is phosphorylated at multiple residues, thereby modifying its biochemical functions, including P53 protein–protein interactions and DNA–protein interactions. Several residues at both the amino and carboxyl terminuses of P53 are phosphorylated or dephosphorylated in response to stress. The stabilization of P53 in cells experiencing stress is crucial for their homeostasis. The phosphorylation is a post-translational modification to P53 that affects its overall appearance and activity. P53 phosphorylation serves as a first focal point in the regulation of P53 activity and the presence of this modification is considered to be a marker of a stress response [12]. The convergence of multiple signals stimulating several P53 phosphorylations is required for a full transcriptional activation response. Following stress, P53 is phosphorylated at multiple residues, thereby modifying its biochemical functions required for increased activity as a transcription factor. To date, nine P53 phosphorylation sites (serine 20, 33, 46, 366, and 392 and threonine 81, 304, 377, and 387) have been detected in specific serine/threonine residues in the N- and C-terminal domains, all of which are able to induce apoptosis. The first crucial step of P53 stabilization is N-terminal phosphorylation at Ser15, Thr18, and Ser20, the latter being linked to P53-MDM2 regulation. Ser15 phosphorylation also triggers a sequential series of additional phosphorylation events in P53 (including phosphorylation of Ser9 -20, -46, and Thr18) that contribute further to P53 induction and activation. Ser15 phosphorylation is therefore an important focal point in the activation of P53 and in the association with the ubiquitin E3 ligases (p300 and MDM2) which mediate the ubiquitylation and proteasomal degradation of P53. In particular, a growing number of studies have indicated that phosphorylation at Ser46 was shown to play a fundamental role in the regulation of P53 apoptotic functions after DNA damage [13,14]. The biological role of the Ser20 site of P53 was identified as the stress-activated Ser20 kinases in the TAD1 transactivation domain [15]. An additional highly conserved phosphorylation site within the P53 C-terminus is the CK2 site on Ser392 whose phosphorylation by casein kinase 2 (CK2) stimulates the sequence-specific DNA-binding function of P53 [15].

P53 can be modified by phosphorylation by a broad range of kinases and the phosphorylation by cell cycle-dependent protein kinases suggests the possibility that the activity of P53 is regulated differentially during the cell cycle. Nevertheless, some studies stated that P53 can also be activated regardless of its phosphorylation state [16].

After activation of P53 in response to a stress signal, the likelihood of P53 binding to P53RE increases. The P53RE motifs are often located at 5′ of the gene or in the first or second intron of the gene regulated by P53. The P53REs have been characterized as the target gene regulatory regions [17]. In light of P53′s ability to bind DNA, the question of whether stress is required to induce binding between P53 and DNA is very interesting. Recent results demonstrate that P53 can bind the DNA of unstressed cells, but the inactivity of P53 is likely due to the repression of MDM2, and MDMX [18].

The homeostasis control of cells exposed to various stresses requires rapid and efficient reprogramming of mRNA translation, to preserve energy for repair and removal of stress damage [19]. Such shifts in stress-responsive protein synthesis the translation of specific mRNAs [20]. At the post-transcriptional level, via mRNA degradation and/or translational repression, more than 60% of total RNAs appear to be regulated by small single-stranded non-coding RNAs indicated as miRNAs (or microRNAs).

Research on miRNAs is one of the most widely discussed topics in science and medicine in the last decade. Bioinformatics tools and high-throughput sequencing contributed to the identification of numerous miRNAs and their potential gene targets. Over the last few decades, miRNAs have become very promising both in the area of biomarkers involved in the onset and progression of diverse biological anomalies and in the area of therapeutic approaches. There has been a drastic surge of interest in miRNA-based therapies developed to either suppress or restore the expression of disease-associated miRNAs. In circumstances where reduced miRNA expression drives the disease, miRNA mimics can be used to restore their expression and function [21]. MiRNAs are highly conserved across many vertebrate genomes [22]. MiRNAs offer the possibility of fast and economical post-transcriptional regulation of gene expression as they simultaneously target multiple transcripts without the need to synthesize proteins [23]. One miRNA regulates multiple mRNA targets, and one mRNA can be regulated by multiple miRNAs, highlighting the complexity of gene regulation by the miRNA network. MiRNAs negatively regulate gene expression by targeting the 3′UTRs of mRNAs. In addition to the cellular environment, there is a large proportion of miRNAs that migrate outside of it and can be found in biofluids including blood (plasma and serum), tears, saliva, urine, colostrum, breast milk, peritoneal fluid, amniotic fluid, cerebrospinal fluid, seminal fluid, synovial fluid, pleural fluid, bronchial lavage, and follicular fluid [24]. A plethora of recent studies has demonstrated that cell-free miRNA, called circulating miRNAs (cmiRNAs), represent the next generation of clinical, non-invasive, biomarkers for many pathologies [25]. In contrast to intracellular miRNAs, cmiRNAs are highly stable due to the kind of packaging that is essential to preventing the digestion of the miRNA by the RNases found in body fluids. Approximately 10% of cmiRNAs are secreted within microparticles such as exosomes, microvesicles, and apoptotic bodies, while the remaining 90% form complexes with RNA ligands such as lipoprotein complexes like high-density lipoprotein (HDL), Argonaute2 (Ago2) and nucleophosmin I (NPM 1) [21,26].

**Figure 6:**
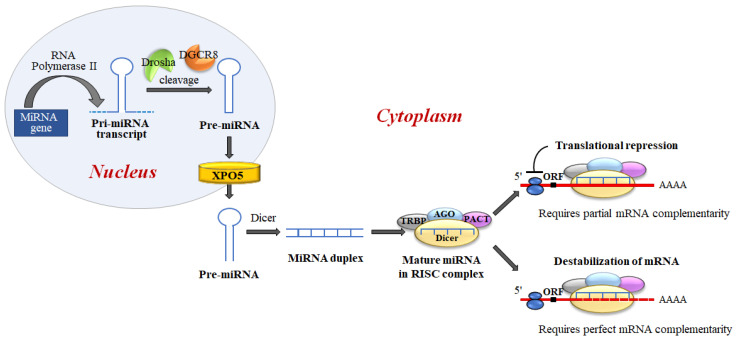
MiRNA biogenesis and miRNA regulation of gene expression. miRNAs are generated, in the nucleus, as primary transcripts (pri-miRNA) by RNA polymerase II/III. The miRNA precursor (pre-miRNA) is processed by Drosha, an RNase III enzyme, and by two molecules of a protein known as Pasha (DGCR8). DGCR8 contains an RNA-binding domain that binds the primary pri-miRNA to stabilize it for processing by Drosha. Drosha and DGCR8 homeostatically control miRNA biogenesis by an auto-feedback loop. After processing, pre-miRNAs, of approximately ~60–80 nt, are exported into the cytoplasm by Exportin-5 (XPO5). Next, the mature miRNA released by the Dicer endonuclease is assembled into the RNA-induced silencing complex (RISC). RISC directs its bound miRNA to untranslated regions of the partially complementary 3′mRNA, resulting in translation inhibition or targeted destruction of the mRNA. MiRNAs regulate target mRNA expression through two major mechanisms: translation blocking in the initiation step or the elongation phase; and removal of the polyA tail promoting deadenylase activity followed by mRNA degradation.

MiRNAs biogenesis is a complex process, which includes several crucial steps including transcription of primary (pri)-miRNAs; cleavage of pri-miRNAs to form precursor (pre)-miRNAs; and finally, cleavage of pre-miRNAs to form mature miRNAs [27] (Figure 6).

Disturbance of cellular homeostasis can interrupt each of the steps of miRNA biogenesis, leading to deregulation of the elaborate miRNA-controlled gene expression pathways, with the result that the cell is more susceptible to stress and its damaging outcomes. Emerging evidence suggests that stress directly influences not only miRNA quantity but also miRNA biogenesis [28,29,30,31].

In this complex network, miRNAs interact at multiple levels and the critical role that miRNAs play in the regulation of stress responses has only recently gained significant attention [29]. Interestingly, P53 regulates the expression of numerous miRNAs and in turn, it is a target of a set of miRNAs that represses P53 protein level and function, by forming a feedback loop with P53. MiRNAs can negatively regulate P53 protein levels through direct repression of P53 expression, or positively regulate P53 activity through the repression of several negative regulators of P53 [32].

Stress-induced variations in miRNA expression levels may partly explain the “stress hardening” phenomenon, clarified by Kultz et al. (2005) [33], in which miRNAs ensure the buffer of transcriptional peaks while maintaining a constant physiological level of gene expression [34]. This concept can be translated to stress resilience, in which the preconditioning of one stress increases tolerance to another stress [29]. As circulating miRNAs have a long half-life, their constant presence from the first exposure to a stressor allows the miRNA-mediated gene expression regulation to cope with future stressors exposure. However, stress is not always recoverable physiologically. If the stress becomes severe, it can induce changes in the miRNA sequence, modifications of miRNA end, “strand switching” [35] or the non-translating mRNAs and their RNA-binding proteins, aggregate into ribonucleoprotein granules to protect cellular mRNAs.

## 4. MiRNA Interacts with P53 at Multiple Levels

In recent years, the important role of cross-talk between P53 and microRNAs in physiological stress signaling has been emphasized. In this paragraph, we illustrate that miRNAs interact with P53 at multiple levels, while P53 is able to regulate the transcription expression and the maturation of a group of miRNAs. On the other hand, miRNAs can regulate P53 activity and function through direct repression of P53 or its regulators. This elaborate regulatory scheme emphasizes the relevance of miRNAs in the P53 network and vice versa. Based on studies to date we discuss three-hit hypotheses of regulatory cross-talk between P53 and miRNAs:

Hit 1 MiRNAs directly control the level of P53 protein.
Hit 2 P53 controls the expression level of some miRNAs through the regulation of their transcription or biogenesis.
Hit 3 MiRNAs indirectly control the level of P53 protein by targeting its regulators (such as MDMs).

### 4.1. MiRNAs Directly Control the P53 Protein Level (Hit 1)

MiRNA interacts directly with P53 mRNA, binding to the 3′-UTR and hindering the translation of the mRNA target and downregulating the P53 protein level. Several miRNAs including miR-125b, miR-504, miR-25, miR-30d, miR-34a, miR-122, miR-29, miR-192, miR-194, and miR-215 have been shown to regulate P53 abundance and/or activity. These miRNAs bind to 3′-UTR of the P53 mRNA and downregulate P53 level and function. MiR-125b and miR-504 are the first two miRNAs that have been identified to directly target P53 [36]. MiR-125b is a miRNA highly expressed in the brain, and also acts as a negative regulator of P53 in zebrafish. Over-expression of miR-125b reduces the endogenous levels of P53 protein and reduces apoptosis, whereas knockdown of miR-125b increases P53 protein level and induces apoptosis in human cells and zebrafish.

These results show that miR-125b is an important negative regulator of P53 and P53-induced apoptosis during development and the stress response [32]. In addition to directly regulating P53, miR-125b controls several factors in the P53 pathway, including apoptosis such as PUMA, insulin-like growth factor-binding protein 3 (IGFBP3), and BCL2-antagonist/killer 1 (BAK1) or cell cycle regulators such as cyclin C, CDC25C and cyclin-dependent kinase inhibitor 2C (CDKN2C). By doing this, miR-125b can regulate and buffer the P53 pathway [37]. A study conducted by Hu W et al. (2010) [38], using a bioinformatic approach, identifies some P53-regulating miRNAs. MiR-504 is one of the first miRNAs identified P53-targeting. MiR-504 reduces P53 protein levels by targeting two binding sites in the 3′UTR of P53 mRNA. In response to stress, miR-504 is over-expressed to reduce P53 abundance. The role of miR-25 and miR-30d, revealed later in time, appears to downregulate P53 protein levels as well as reduce the expression of P53 transactivated genes. Over-expression of these miRNAs inhibits P53-mediated apoptosis, cell cycle arrest, and senescence. Conversely, inhibition of these miRNAs induces increased P53 expression and elevated apoptosis in several cell lines [39].

The MiR-29 family directly regulates the P53 level. Data gathered by Park et al. showed that the over-expression of mcoriR-29 downregulates cell division cycle 42 (CDC42) and p85α, which in turn leads to activation of P53. CDC42 is a protein involved in the cell cycle control which regulates signaling pathways involved in several cellular functions including cell morphology, cell migration, endocytosis, and cell cycle progression. p85α protein is the regulatory subunit of phosphatidylinositol-3 kinase (PI3K) and plays a major role in maintaining the balance of cellular survival and apoptosis. Park et al. showed that p85α had two miR-29 binding sites in its 3′-UTR region [40]. Similarly, miR-98, miR-150, miR-214, miR-375, miR-19b, miR-1285, and miR-3151 decreased P53 protein levels by binding the 3′UTR of P53 and affecting P53 function in the cell cycle arrest, apoptosis, endogenous regulation of stress resistance, and homeostasis maintenance [10].

Many miR-25s target mRNAs are involved in biological processes such as cell proliferation, differentiation, migration, apoptosis, oxidative stress, and help adaptation to environmental fluctuations. Therefore, it is no surprise that miR-25 has been reported as a key regulator of the common physiological state of stress. Furthermore, miR-25 was identified as a miRNA repressed indirectly by P53 through the transcriptional regulators of the *TP53* gene, E2F1 (also called retinoblastoma binding protein-3) and MYC; in this regard, there are experimental data only concerning the pathological state of glioblastoma [41]. MiR-25 is a negative regulator of the P53 pathway leading to inhibition of cellular proliferation by arresting the cell cycle.

A set of miRNAs directly targets P53 to repress the protein level by controlling its accumulation and, in turn, the same miRNAs are regulated by P53 through transcriptional activation or repression. There is an autoregulatory circuit involving the expression of P53 to regulate the expression of certain miRNAs and, in turn, the upregulation of certain miRNAs buffers the transcription spike of P53; the balance of these two opposing molecular signals mediates P53 preconditioning to the recovery of homeostasis. There is a recurrent autoregulatory circuit involving the expression of P53 to regulate the expression of some miRNAs and, as feedback, the upregulation of some miRNAs buffers the P53 transcription spike. The balance of these two opposing molecular signals could mediate P53 preconditioning to the recovery of the homeostasis [42]. Negative feedback regulation between miRNAs and P53 has also been reported recently about miR-25, miR-29, miR-18b, and miR-122. MiR-122 is post-transcriptionally upregulated by P53 and, in turn, suppresses P53 activity thus forming a negative feedback loop [43]. Interestingly, it was found to be post-transcriptionally upregulated by P53 and in turn suppress P53 activity, thus forming a negative feedback loop.

### 4.2. P53 Controls the Expression Level of Some miRNAs through the Regulation of Their Transcription or Biogenesis (Hit 2)

As a transcription factor, P53 not only regulates the expression of protein-coding target genes but may also play a key role in the regulation of miRNA involved in the P53 pathway [10]. P53 can both activate and repress miRNA expression by acting as a transactivator of oncosuppressive miRNAs and as a repressor of oncogenic miRNAs. MiRNAs upregulated by P53 often target anti-apoptotic and pro-proliferative genes, thus reinforcing the function of P53, or are involved in negative feedback circuits between miRNAs and P53 [39].

The first significant induction by P53 was observed for the miR-34 family. In addition to being a direct P53 target, it regulates the expression of many genes involved in the P53 pathway and has a negative feedback regulation with P53. In mammals, the miR-34 family is composed of three miRNAs encoded by two loci: miR-34a is transcribed independently, whereas miR-34b and miR-34c share a primary transcript. All three members of the miR-34 family have P53-responsive elements in their promoter regions. In mammals, the miR-34 family is linked to diverse developmental processes, such as cell-cycle arrest, apoptosis, lipid metabolism, metabolic homeostasis, and insulin secretion [44]. This miRNA family plays a key role in mediating the anti-proliferative and pro-apoptotic effects of P53 by targeting cell cycle genes and proto-oncogenes. The change in the level of miR-34 can cause reprogramming of gene expression: the increase in miR-34 induces cell apoptosis, while the decrease in miR-34 leads to a prolongation of cell survival [39]. miR-34a is involved in cell cycle arrest by targeting cyclins and cyclins dependent kinases (CDKs) such as CDK4 and CDK6, cyclin D1, and cyclin E2, which have roles in the G1/S transition.

**Figure 7:**
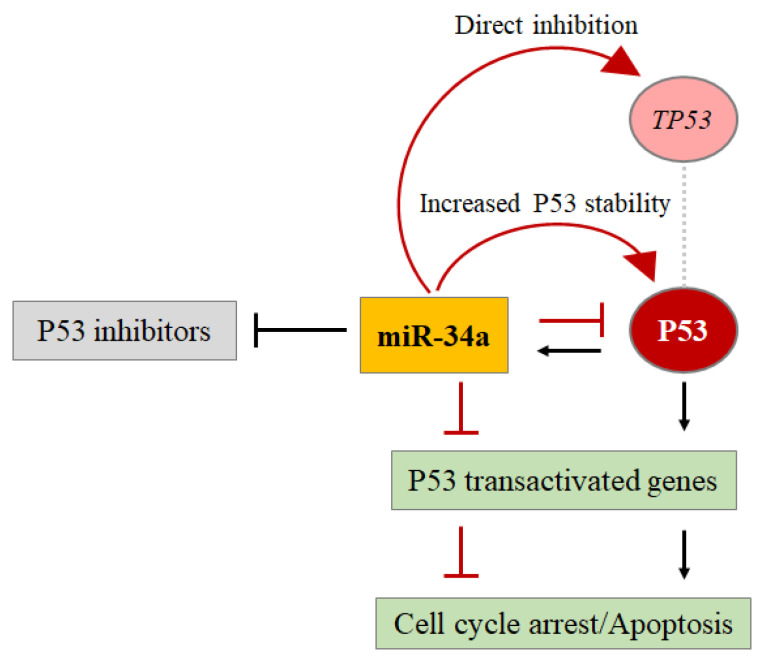
The recurrent autoregulatory circuit between miR-34a and P53. P53 regulates the expression of miR-34a which regulates P53.

Additional anti-proliferative effects of miR-34a are reached by the repression of growth factor receptor tyrosine kinases (RTKs) and their downstream kinase pathways. miR-34a directs the cell towards apoptosis, targeting the *BCL2* oncogene. In turn, BCL2 inhibits BH3 pro-apoptotic proteins, such as PUMA, blocking the activation of the intrinsic apoptotic pathway [5]. Navarro and Lieberman advanced an interesting theory on the regulation between miR-34a and P53 explaining that competing negative and positive feedback loops determine the net effect of miR-34a on P53 function. The authors argue that the activation of P53 by cellular stress leads to the transcription of miR-34 miRNAs. This miRNA improves P53 function via inhibition of multiple negative regulators to increase P53 transcriptional activity, an increase in protein stability (miR-34a feed-forward loop), inhibition of protein function, and direct inhibition of *TP53* or many P53-activated genes (negative feedback loop). The net effect of miR-34a on the P53 response depends on the relative importance of these pathways, which is determined, under physiological conditions, by differences in cell gene expression concerning the organism and the living environment [45] (Figure 7).

Recent research has shown that the miR-34 family is crucial in stress response fine-tuning and especially to individual stress reactivity. Mice carrying a genetic deletion of all miR-34 isoforms (triple knockout, TKO) lack the stress-induced serotonin (5-HT) and GABA release from the medial prefrontal cortex (mpFC) and basolateral amygdala (BLA). miR-34 appears to modulate the mpFC 5-HT/BLA GABA response to stress by acting on CRFR1 in the DRN determining the development of individual coping strategies to stress [46].

To date, mir-34 is considered the major regulator of stress response pathways recoverable in physiological conditions, because its expression, strictly regulated by P53, controls gene expression programs that improve adaptation to stressful environments [44].

Interestingly, P53 can transcriptionally activate other miRNAs with antiproliferative activities (such as miR-15a/16, miR-23a, miR-29, miR-107, miR-143, miR-145, miR-192, miR-194, miR-215, miR-605, let-7) and transcriptionally repress miR-17-92 cluster and miR-221/222. For instance, miR-15a/16, miR-29, miR-192, miR-215, and let7 target the *BCL2* oncogene controlling DNA damage-induced apoptosis [47]. Some of these miRNAs are involved in a negative feedback loop with P53 [10,23].

It has to be stressed that, through targeting cell cycle and proliferation pathways, all these miRNAs support the growth-suppressive function of P53 under stress conditions. However, direct links between stress-induced P53 activation and miRNA up-regulation have been featured mainly for miR-34a as well as for miR-143 and miR-145 [23]. MiR-143 and miR-145, which belong to the same miRNA cluster, are post-transcriptionally activated by upregulated P53 generating a miRNAs–MDM2–P53 feedback loop that negatively modulates the expression of the MDM2 target gene. The miRNA-dependent MDM2 turnover contributes to the equilibrium of repeated P53 pulses in response to stress damage [48]. Elevated levels of miR-143 and miR-145 can induce apoptosis of wild-type P53 HNSCC cells and can suppress cell proliferation in vitro and in vivo by downregulating MDM2 and upregulating P53 expression. However, miR-143/145, MDM2 and P53 form a regulatory circuit that helps control cell proliferation and apoptosis.

In humans, miR-103 and -107, occupy different chromosomes and differ only by one nucleotide near their 3’ ends. Their host genes are conserved in all known vertebrates, and act fundamental and similar roles in the control of stress and apoptosis, especially at the level of the endoplasmic reticulum (ER). Over-expression of miR-103/107 gained via miR-103/107 mimics promotes ER stress and apoptosis, whereas, in contrast, knockdown of miR-103/107 via the miR-103/107 inhibitor suppresses ER stress and apoptosis [49]. The modulatory role of P53 as a regulator of specific miRNA maturation, including miR-15a, miR-16, miR-143, miR-145, miR-199a, and miR-122 is noteworthy. These miRNAs negatively modulate the expression of genes involved in the cell cycle and cell proliferation such as *KRAS* and *CDK6*.

Interestingly, under prolonged stress, P53 binding to an upstream enhancer of let-7a and let-7b, which likely competitively precludes the binding of other transcriptional activators, resulting in let-7 family downregulation. Reduced expression of let-7 is associated with tumor malignant phenotypes. The let-7 family is tumor-suppressive miRNA that targets growth-related genes such as *RAS*, *CDC25A*, and *CCND1*. Let-7 miRNA levels inversely correlated with protein expression of several key oncogenes, such as *RAS* and *MYC*, but not its mRNA levels, suggesting a mechanism of let-7-mediated translational inhibition without mRNA degradation [39].

It is generally accepted that P53 acts primarily as an activator of the induction of tumor-suppressive miRNAs; however, recent studies suggest that P53 may repress transcription of oncogenic miRNAs, although this occurs less frequently. For instance, P53 inhibits miR-224, miR-502, and the miR-17-92 cluster expression by binding to their promoter to inhibit cell proliferation [50,51].

Owing to its vital regulation, the role of P53 in the miRNA network has been extensively explored over the past decade and has recently been joined by the role of P53 in the maturation process of miRNAs. During the miRNA maturation process, P53 cooperates with the Drosha processing complex, by association with DEAD-box RNA helicase p68 (DDX5), by supporting Drosha-mediated editing of the pri-miRNA to precursor miRNAs. Additionally, P53 regulates the selection of the specific target of the miRNAs inducing RNA-binding-motif protein 38 (RBM38), which binds to 3′UTR of mRNAs and blocks the access of miRNAs to target mRNAs. P53 is also directly associated with the AGO2 to regulate the formation of miRISC [32,52]. The control of the miRNA maturation process strongly influences the recovery of homeostasis during stress. The arrest of miRNA maturation has been shown to cause increased DNA damage and consequently upregulation of P53, emphasizing the reciprocal connection between P53 pathways and miRNAs [53].

### 4.3. MiRNAs Indirectly Control the P53 Protein Level by Targeting Its Regulators (Hit 3)

Recent studies reported that some miRNAs indirectly regulate P53 by interacting with P53-regulating factors. These miRNAs indirectly repress P53, by targeting its negative regulators such as MDM2 and MDM4. The delicate balance between P53, MDM2, and MDM4 is crucial for cell survival. The P53 protein has a short half-life of 6–20 min and the MDM2 protein is the ubiquitin ligase that confers this short half-life [54]. The transition between the right level and P53 activity through MDM2 and MDM4 regulation must be rapid and precise and miRNAs are responsible for maintaining this balance [55]. In homeostatic conditions the P53 levels are kept low; instead, upon cellular stress, high levels of the protein are needed. Upon P53 activation, as a response to a stress signal, P53 expression increases, and it gains the ability to bind to P53-responsive DNA sequence elements.

**Figure 8:**
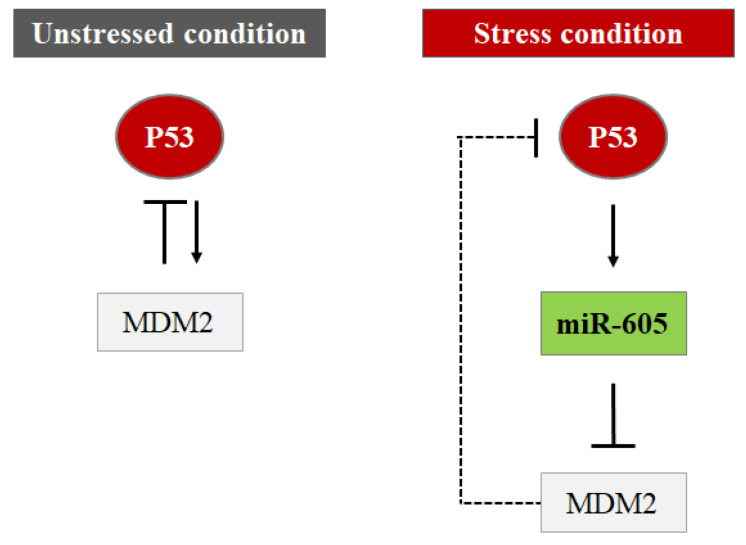
The regulation of MDM2 by miR-605. In normal conditions, P53 activates the transcription of MDM2, which in turn targets P53 for repression, creating a negative feedback loop to inhibit P53 level and function. In response to cellular stress, P53 enhances the miR-605 expression and miR-605 upregulation increases the activity of P53 by repressing MDM2 to remove the inhibitory effects of MDM2 on P53.

As already mentioned, MDM2 is a key repressor of P53 activity; it is also a P53 transcriptional target and creates a negative feedback loop that keeps P53 levels and activity under strict control. Multiple miRNAs can interfere with this P53-MDM2 regulatory circuit by targeting and repressing the MDM2 mRNA to increase P53 activity in cells under stress conditions. Several miRNAs targeting MDM2 have been identified, including miR-192/194/215, miR-143/145, miR-29b, and miR-605. Interestingly, among these miRNAs, miR-192/194/215, miR-143/145, and miR-605 are transcriptionally regulated by P53, and in this way, they form a positive feedback loop with P53. Through this control, P53 promotes the transcription of these miRNAs which in turn target and repress MDM2 resulting in an upregulation of the P53 level. miR-605 can be evaluated as a new regulatory molecule of P53–MDM2 interaction. MiR-605 switches the P53–MDM2 negative feedback loop into a positive feedback loop by promoting a rapid accumulation of P53 to facilitate its function in response to stress. In conditions of physiological stress, the expression of miR-605 is enhanced by P53 activation; consequently, the upregulation of miR-605 increases the transactivation activity of P53 by repressing MDM2 and thereby reducing the inhibition of MDM2 on P53. Through a positive feedback loop, miR-605 allows the rapid accumulation of P53 and cell cycle arrest or apoptosis [56,57,58] (Figure 8).

Additionally, miR25 and miR32 negatively regulate MDM2, and their transcription is negatively regulated by P53 and positively regulated by E2F-1 and MYC (proteins that regulate the passage of a cell from quiescence into the cell cycle). miR-25 and miR-32 bind directly to the 3′-UTR mRNA of MDM2 leading to stabilization and increased function of the P53 protein. These microRNAs form feedback loops with MDM2–P53 to decrease MDM2 levels and promote P53 functions.

Cen Zhang et al. (2015) [57] identified miR-339-5p and miR-1827 as miRNAs that activate P53 through binding to the 3′-UTR of MDM2 mRNA. These miRNAs reduce MDM2 protein levels which in turn increase P53 protein levels to promote P53-mediated stress responses.

The miRNAs can regulate P53 activity by targeting MDM4, miR-10a, miR-191-5p, miR-887, miR-661, miR-34a, miR-199a-3p, and let-7 inhibited MDM4 through directly targeting the 3′-UTR of MDM4 mRNA [10]. It is reasonable to assume that miRNAs can target both MDM2 and MDM4 at the same time. Indeed, Hoffman et al. in 2014 [59] identified miR-661 as the miRNA capable of simultaneously targeting MDM2 and MDM4, demonstrating that miR-661, a primate-specific miRNA, simultaneously targets MDM2 and MDM4 by binding within Alu elements in their 3′UTRs, increasing the functionality of P53. In this way, even very modest effects of miR-661 on each of these two proteins can only result in a significant effect on cellular P53 activity. The conclusion is that in physiological conditions miR-661 increases the functionality of P53 by suppressing tumor pathologies, on the contrary in pathological conditions with *TP53* mutations, miR-661 can become pro-oncogenic.

A key role is covered by miR-34a, already seen in hit 2, which positively regulates P53 protein levels by targeting not only MDM4 but also SIRT1, HDAC1, and YY1. SIRT1 controls P53-dependent apoptosis through P53 acetylation and destabilization, while YY1 downregulates P53 by stimulating its ubiquitination and degradation [39]. miR-34a as the P53 target gene forms a positive feedback loop with P53 in which P53 induces miR-34a expression that in turn upregulates P53 by suppressing SIRT1 and YY1. These miRNAs are joined by miR-29 family members, so miR-29 down-regulates p85 alpha, a regulatory subunit of PI3 kinase, and thereby enhances P53 activity through the negative loop between PI3K–AKT–MDM2 and P53. The miR-29 targets two other P53 negative regulators, CDC42 and PPM1D phosphatase, to increase P53 levels and activity. In addition, miR-29 is a P53 inducible miRNA.

Several ribosomal proteins (RPs) have been shown to bind to MDM2 and inhibit MDM2 E3 ligase activity, leading to P53 stabilization and cell cycle arrest, thus revealing an RP–MDM2–P53 signaling pathway that is critical for ribosome biogenesis surveillance. The miR-542-3p regulates MDM2 by targeting the ribosomal protein RPS23 to upregulate RPL11, thereby sequestering MDM2 and inhibiting MDM2-mediated P53 degradation; consequently, P53 results as stabilized and activated [10].

In the mammalian liver, it has been shown that the increase in the level and function of P53 protein can be mediated by miR-122, which by directly targeting the 3′UTR of cyclin G1 represses cyclin G1 by downregulating MDM2 and increasing the level and activity of P53. cyclin G1 forms a complex with phosphatase 2A (PP2A). PP2A removes a phosphate group from phosphorylated MDM2 leading to more functional MDM2 because phosphorylated is less active. In contrast, cyclin G1 downregulation, by inhibiting PP2A, induces phosphorylated MDM2 upregulation, which is less functional [39].

**Figure 9:**
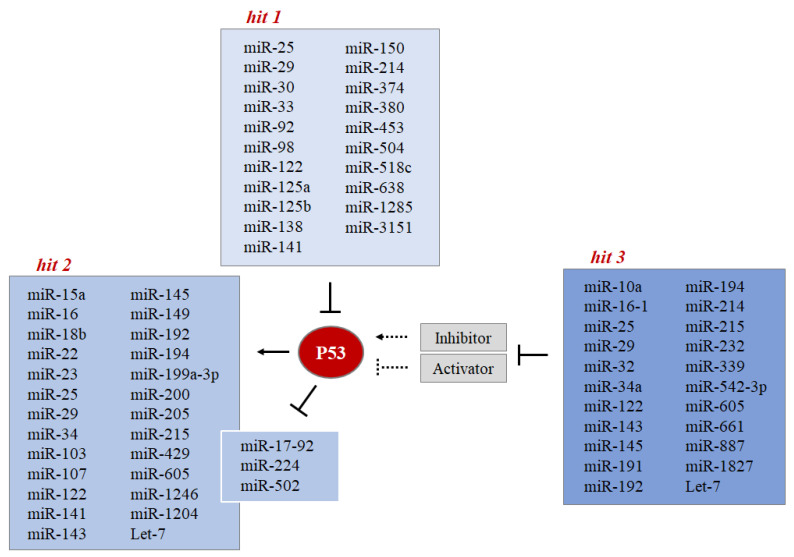
Schematic mechanism of P53–miRNA interaction at multiple levels. MiRNAs directly target P53 by binding the 3′UTR of P53 mRNA. The interaction downregulates the translation of P53 mRNA (hit 1). In a regulatory circuit P53 increases or decreases the expression of miRNAs by inhibiting regulators (hit 2). Furthermore, some miRNAs targeting P53 regulators vary P53 outcomes (hit 3). If the regulators are positive, P53 outcomes culminate in decreases, or, on the other hand, negative regulators lead to a P53 increase.

In the latter part of the review, we showed how cross-talk between miRNAs and P53 controls P53 expression and activity and how, to regulate P53 and its pathway, miRNAs employ different mechanisms to do so. These mechanisms may act not only by directly regulating P53 but also through the control of its negative–positive regulators. This constant surveillance maintains P53 low levels, under normal cellular conditions, and increases P53 levels or activity in response to cellular stress. The ultimate goal of this sophisticated regulation is to make P53 able to rapidly increase to mediate cell cycle arrest or, in case of excessive cell damage, induce apoptosis (Figure 9).

## 5. Conclusions

After decades of research, the role of P53 in controlling specific responses to stress remains an enigma. Scientific research has devoted much interest to mechanisms linking the role of P53 with severe stress, where P53 permanently deregulates the system, leading to cancer, neurodegeneration, or pathogen infection. While little has been described of the P53 ability to restore cellular homeostasis during physiological stress. To date, most of the P53 post-transcriptional and post-translational regulatory mechanisms and their cooperation remain open areas of investigation. In this review, we wanted to pay special attention to the dialogue between P53 and its regulatory mechanisms during mild stress.

We sought to presume how stress signaling alters the function of miRNA-bioprocessing core components linked to P53 leading to the recovery of cellular homeostasis. If the stress continues over time or stress intensity increases the consequences may include a vicious cycle, whereby stress impairs miRNA processing which in turn makes cells even more prone to stress [30]. Undoubtedly, the next decade will see a huge expansion of research on the role of P53 and related miRNAs, as key epigenetic regulators in the physiological recovery of stress related to changes in the living environment. Until now, the role of P53 has been mainly considered in relation to the tumor and inflammatory pathologies and the cross-talk between miRNA and P53 has been examined to identify a biomarker or a therapeutic choice. Our interest in this topic arose to fill the desire to consolidate the hypothesis that P53 during mild stress could be studied to improve performance and therefore resilience in working animals or athletes in order to prevent performance losses and psychological or physical problems. We hope that future molecular studies will investigate the mechanisms that regulate physiological stress and stress resilience to improve the way we manage the daily well-being of animals and promote healthy lives for them.
